# Effect of using cardiovascular risk scoring in routine risk assessment in primary prevention of cardiovascular disease: an overview of systematic reviews

**DOI:** 10.1186/s12872-018-0990-2

**Published:** 2019-01-09

**Authors:** Krzysztof Studziński, Tomasz Tomasik, Janusz Krzysztoń, Jacek Jóźwiak, Adam Windak

**Affiliations:** 1 Department of Family Medicine, Chair of Internal Medicine and Gerontology, Jagiellonian University Medical College, 4 Bochenska Street, 31-061 Krakow, Poland; 2 Department of Family Medicine and Public Health, Institute of Medicine, University of Opole, Opole, Poland; 3 College of Family Physicians in Poland, Warszawa, Poland

**Keywords:** Cardiovascular diseases, Primary prevention, Risk assessment, Cardiovascular system, Risk factors

## Abstract

**Background:**

Our objectives were to critically appraise and summarise the current evidence for the effectiveness of using cardiovascular disease (CVD) risk scoring (total risk assessment - TRA) in routine risk assessment in primary prevention of CVD compared with standard care with regards to patients outcomes, clinical risk factor levels, medication prescribing, and adverse effects.

**Methods:**

We carried out an overview of existing systematic reviews (SRs). Presentation of the results aligned guidelines from the PRISMA statement. The data is presented as a narrative synthesis. We searched MEDLINE (Ovid), EMBASE, CENTRAL and SCOPUS databases from January 1990 to March 2017, reviewed the reference lists of all included SRs and searched for ongoing SRs in PROSPERO database. We encompassed SRs and meta-analyses which took into account RCTs, quasi-RCTs, and observational studies investigating the effect of using CVD risk scoring. Only studies performed in a primary care setting, with adult participants free of clinical CVD were eligible. Intervention was CVD risk assessment with use of the total CVD risk scoring compared with standard care with no use of TRA .

**Results:**

We identified 2157 records, we then recognised and analysed 10 relevant SRs. One SR reported statistically insignificant reduction of CVD death, when using TRA, the second SR presented meta-analysis which reported no effect on fatal and non-fatal CV events compared with conventional care (5.4% vs 5.3%; RR 1.01, 95% CI 0.95 to 1.08; I^2^ = 25%). Three SRs have shown that using TRA causes no adverse events. The impact of TRA on global CVD risk as well as individual risk factors is ambiguous, but a tendency towards slight reduction of blood pressure, total cholesterol and smoking levels, especially in high risk patient groups was observed. TRA had no influence on lifestyle behaviour.

**Conclusions:**

There is limited evidence, of low overall quality, suggesting a possible lack of effectiveness of TRA in reducing CVD events and mortality, as well as a clinically insignificant influence on individual risk factor levels. Using TRA does not cause harm to patients.

**Trial registration:**

Systematic review protocol was registered with the International PROSPERO database - registration number CRD42016046898.

**Electronic supplementary material:**

The online version of this article (10.1186/s12872-018-0990-2) contains supplementary material, which is available to authorized users.

## Introduction

Cardiovascular disease (CVD) is presently the leading cause of mortality, morbidity and disability worldwide [1, 2]. Well-established, modifiable CVD risk factors include: elevated blood pressure, hypercholesterolaemia, diabetes, lack of physical activity, obesity, inappropriate diet, and smoking [3, 4].

In 1961, Kannel et al. [5] used the term “factors of risk” for the first time and proved that hypertension, hypercholesterolaemia and other risk factors precede the development of coronary heart disease [CHD] in humans.

Understanding CVD risk factors makes prevention possible. Risk factor modification can reduce the number of premature deaths as well as the number of other clinical events, both in people without established CVD (primary prevention) and people with confirmed CVD (secondary prevention) [6].

Following Kannel’s work, a new concept of the absolute/global/total CVD risk has become widely adopted. By definition, absolute CVD risk is the actual risk of developing the disease within a defined population in a defined period of time (typically 5 or 10 years) [7]. The absolute CVD risk is calculated by using a combination of major risk factors. Risk score algorithms incorporating multiple risk factors are used to calculate absolute risk for an individual patient [8].

The number of risk scores developed in European countries, USA and other parts of the world have grown rapidly. In 2016, Damen et al. [9] identified 363 prognostic models or risk-scoring methods with potential use in targeting primary prevention of CVD. Contemporarily, the most well-known and widely used are: Systematic COronary Risk Evaluation (SCORE) algorithm [10], QRISK2 [11], the World Health Organization (WHO) risk score, and the American College of Cardiology/American Heart Association (ACC/AHA) 2013 Pooled Cohort risk equations [12].

Presently, major clinical practice guidelines (CCS [13], ESC/ESH [6], ACC/AHA [14], JBS3 [15]) recommend assessing risk of CVD using the absolute/global/total CVD risk scores. It is stated that the use of CVD risk scores increases the accuracy of prediction of CVD events and guides management decisions in primary prevention.

The effectiveness of using absolute/global/total CVD risk scores called also total risk assessment (TRA) has been assessed by numerous randomised control trials (RCTs), published over the last two decades. The research community also tried to summarise existing evidence in this field, which resulted in the publication of a few systematic reviews (SRs) [16–19]. However, despite numerous studies, there are still some unsolved issues. One of them is whether using TRA is clinically effective when important outcomes for patients are taken into account (e.g. mortality, CV events). Even when TRA appropriately predicts CV events, it does not mean that beneficial clinical effects will occur. Furthermore, there is a lack of data about the potential adverse effects of TRA. The absence of information does not mean that this procedure is completely safe.

Summing up, the effectiveness of using TRA in clinical practice is still poorly understood. Although it may be beneficial and improve health (e.g. identifying high-risk individuals who will most likely benefit from risk factor management), it may also be harmful (e.g. undertreatment of the youth, overtreatment of the elderly) and lead to misuse of resources (e.g. time and cost of laboratory tests).

## Objectives

This study has the following objectives: (1) to critically apprise and summarise the best current evidence for the effectiveness of using TRA in routine risk assessment in primary prevention of CVD compared with standard care; (2) to assess whether use of a particular risk score followed by structured or unstructured intervention is more effective than any other risk score in improving patient outcomes and (3) to discuss how our findings can be used to guide clinicians and policymakers, and provide a guideline for future authors.

## Methods

### Protocol and registration

Our systematic review protocol was registered with the International Prospective Register of Systematic Reviews (PROSPERO) on 01 September 2016 (registration number CRD42016046898). The full version of the protocol was later published in the BMJ Open [20]. Any deviations from this plan, which were minor, are described in Additional file 1.

### Eligibility criteria

Studies were selected according to the prespecified criteria defined below. Those criteria were also mentioned in detail in the full version of the protocol of our study published in the BMJ Open [20].

#### Study design

We performed an overview of SRs and meta-analyses which took into account RCTs, quasi-RCTs, and observational studies investigating the effect of using CVD risk scoring in routine risk assessment in primary prevention of CVD.

#### Participants

Only studies with adult participants (19 years of age and over) and free of clinical CVD, were eligible. Studied patients may have had different CVD risk factors or other diseases including diabetes and chronic kidney disease.

#### Intervention

CVD risk assessment with use of total risk assessment (TRA), performed by physicians or other healthcare professionals.

#### Comparator

Standard care with no use of the global CVD risk scoring provided by a physician or healthcare professional.

#### Outcomes

##### Primary outcomes

(1) CVD death, (2) Fatal and non-fatal cardiovascular events, (3) Adverse events (any physical, psychological or social events).

##### Secondary outcomes

(1) All-cause mortality, (2) Change in predicted global CVD risk, (3) Change in patient CVD risk factors - change in blood pressure, cholesterol level, smoking, exercise, diet, alcohol consumption and obesity, (4) Prescription of risk-reducing drugs according to prevailing guidelines (aspirin, antihypertensives, lipid-lowering drugs), (5) Pharmacotherapy without or against current clinical guideline recommendations.

Adverse events mentioned as primary outcomes could be as follows: (1) physical – e.g. hypertension or dyslipidaemia complications in young patients who were excluded from pharmacotherapy due to low CVD risk score; (2) psychological – e.g. anxiety, depression, stress caused by diagnosis and being labelled as “chronically ill”; (3) social – e.g. cost and additional time spent on unnecessary consultations, role changing in family or society.

#### Setting

Only studies performed in an outpatient setting were eligible since TRA guides management decision in primary prevention in patients without known CVD.

### Search strategy

We searched MEDLINE (Ovid), EMBASE, CENTRAL (Cochrane Central Register of Controlled Trials) and SCOPUS databases from January 1990 to March 2017. All databases from 1990 onwards were scanned because only a very small number of systematic reviews had been conducted before that time [21, 22]. The search was limited to English language literature. It was supplemented by a search for unpublished, ongoing, or recently completed systematic reviews in PROSPERO database. In addition, we reviewed the reference lists of all included systematic reviews. Our detailed search strategy for MEDLINE is presented in Additional file 2.

### Data management

All identified records were uploaded or manually entered into Mendeley v1.17.6 (Elsevier). After duplicate removal, titles and abstracts from the searches were independently screened by two authors. Full-text articles were retrieved for all potentially includable SRs. Any disagreements were resolved through discussion. In case of lack of consensus, a third author was arbitrated.

The methodology for data extraction and analysis was based on guidance from Preferred Reporting Items for Systematic Reviews and Meta-Analyses (PRISMA) statement [23] and the Cochrane Handbook of Systematic Reviews of Interventions [24]. The PRISMA checklist is presented in an Additional file 3. Two authors independently extracted outcome data from each included SR using a predefined data extraction form. Disagreements during the data extraction process were resolved through discussion or arbitrarily by a third author.

We have extracted the following information: (1) administrative and bibliographic data; (2) the characteristics of each review; (3) methodological details and results of meta-bias assessments (if conducted); (4) reported limitations of each review; (5) results and conclusions of the review.

If meta-analysis was presented in one of the included SRs, we extracted both its results and all relevant methodological aspects (e.g. types and unity of data, effect measured, heterogeneity, sensitivity analysis). As we specified in our overview protocol [20], we did not conduct a meta-analysis of meta-analyses, mainly because of the risk of introducing bias. According to Smith et al. [25] and Pieper et al. [26], such undesired bias can be easily incorporated (due to giving excessive statistical power to primary studies included in more than one systematic review) when performing meta-analysis in review of systematic reviews. Additionally, the same authors highlight that there is no well-established quantification method in this field.

Additionally, we looked at the following information: (1) outcomes reported in a particular SR which were recognised by the authors as evidence of the effectiveness of TRA, (2) reported effect of TRA in different populations, (3) reported effect of different TRA in the same population.

### Risk of bias in included systematic reviews

Two reviewers independently appraised risk of bias of the included systematic reviews using a validated assessment of multiple systematic reviews (AMSTAR) checklist, which is the most commonly employed method to assess the quality of systematic reviews included in overviews [27]. We scored each systematic review with a maximum of 11 points. Disagreements were resolved through discussion or arbitrarily by a third author. Each systematic review was assigned to one of three quality levels (0–3 points - low quality, 4–7 points - medium quality and 8–11 points - high quality) [28, 29].

### Risk of bias across systematic reviews

To assess selective outcome reporting within systematic reviews, we compared those which were planned to be assessed in the systematic review protocols (or, if unavailable, in the methods section of the published report of SR) and other outcomes reported in the results section of the published report of SR. To minimise publication bias, we identified all relevant ongoing SRs by searching the PROSPERO database. To assess the degree of overlap in the inclusion of primary studies between systematic reviews, the citation matrix was generated by one reviewer and checked by a second for accuracy (Additional file 4). The degree of overlap was calculated with use of the corrected cover area (CCA) in our citation matrix by an experienced statistician [26].

## Results

**Fig. 1:**
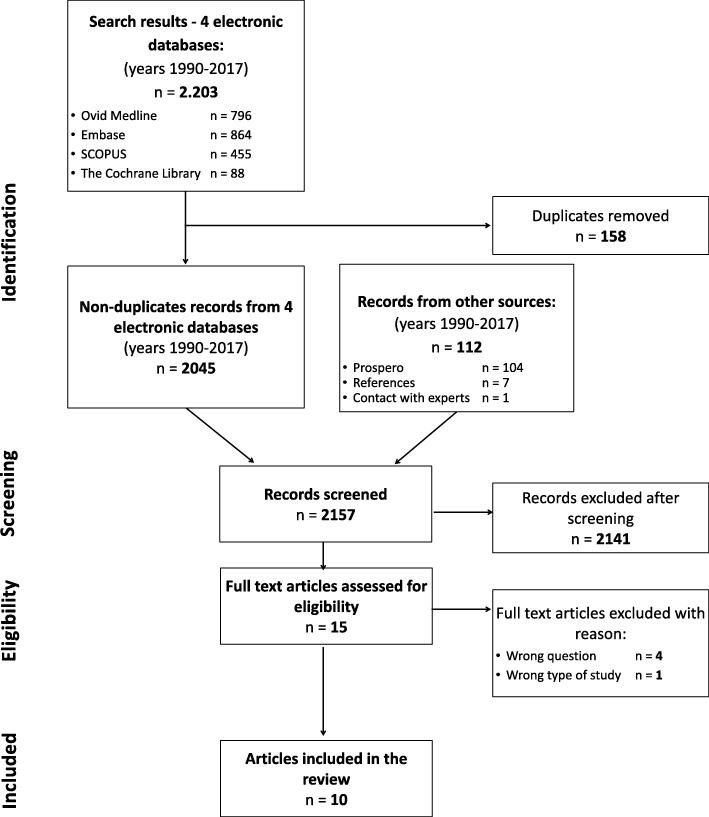
Flow diagram

After duplicates were removed, the electronic search identified 2045 papers. One hundred twelve records from other sources were added (104 records of ongoing or recently completed SRs from PROSPERO database, 7 papers found in the reference sections of known SRs and one received due to contact with the authors). 2157 records were independently screened by two authors. Fifteen full-text articles were screened for eligibility, of which 4 were not related to the effectiveness of TRA [30–33], 1 was not SR [34], and the remaining 10 SRs included 66 unique primary studies and were included (see Additional file 4). Flow diagram is presented at Fig. 1.

### Summary of included systematic reviews

Characteristics of included systematic reviews are presented in Table 1.

**Table 1:** Characteristics of included systematic reviews

Author, Date	Research objectives / questions	Population	Intervention	Comparison	Outcomes		Quality of evidence of primary studies as reported by authors
					Primary and secondary	Harmful specified	
Brindle 2006 [16]	The impact of assessing CVD risk (in the primary prevention of CVD) on clinical outcomes	Any age, free from symptomatic CVD. Patients with diabetes, raised risk factors or preventive treatment eligible	Use of a cardiovascular risk score by healthcare professional	Usual care with appropriate treatment and lifestyle recommendations	(1) CVD or CHD fatal or non-fatal events, (2) risk factor levels, (3) absolute CVD/CHD risk, (4) the prescription of drugs, (5) changes in health-related behaviour.	No	Unclear
Sheridan 2008 [17]	Whether knowledge of a global CHD risk scores translate into clinical benefits and whether there are any harms	Adults (> 18 years old) with no prior history of CVD	Providing physicians with global CVD risk scores or allowing them to calculate it themselves	No information about global CVD risk	(1) Improved physicians’ adherence to guidelines, (2) increased appropriate prescribing, (3) increased patient acceptance/ adherence to therapies, (4) improved control of CVD risk factors, (5) a reduction in CVD events.	Any adverse physical or psychosocial outcome	6 fair, 5 methodo-logically limited studies
Sheridan 2010 [18]	The effect of providing global CHD risk information to adults	Adults with no history of CVD	Global CHD risk presentation to the patient as the primary intervention or part of a multipart intervention	Not specified	(1) Accuracy of risk perception, (2) intent to start treatment, (3) adherence to therapy, (4) change in predicted global risk or event rates, (5) changes in patient BP, cholesterol levels, aspirin use, smoking cessation, diet or exercise.	No	6 good, 12 - fair quality studies
Waldron 2011 [35]	The effectiveness of different interventions used to communicate CVD risk and the impact of the formats used	Adults (> 18 years old), primary or secondary care	Risk communication interventions (of any format) for individualised cardiovascular risk assessment	Other interventions, or usual care.	Understanding, affect, intention to modify behaviour and reduction in actual risk	No	2 – good, 2 medium quality studies
van Dieren 2012 [36]	Impact assessment of a CVD risk model, scores or rules, applied to patients with type 2 diabetes. (Also models development and validation studies)	People with type 2 diabetes and general population but included diabetes as a predictor	Risk assessment and format of its communication	Not specified	Not specified	No	Not reported
Willis 2012 [19]	The effectiveness of the use of CVD risk scores when combined with lifestyle interventions in the prevention of CVD	High risk patients, aged ≥40 and free from CVD	CVD risk assessment using validated risk scores followed by multifactorial interventions	Not specified	(1) CVD mortality, (2) incidence of CV events, (3) changes in a validated CVD risk score.	No	3 high, 1 moderate, 1 low quality study
Usher-Smith 2015 [37]	Whether the provision of information on CVD risk impacts decision-making, behaviour and patient health	Patients with no history of CVD	Provision of a CVD risk model estimate to either patients or practitioners	Other interventions, such as lifestyle advice or exercise programmes	Risk perception; changes in: health-related behaviour, BP, cholesterol levels, modelled cardiovascular risk, medication prescribing, anxiety and psychological well-being, contact with healthcare professionals after provision of risk information	No	5 low; 1 low-medium; 7 medium; 3 medium-high; 1 high quality study
Tomasik 2017 [38]	The effects of global CVD risk estimation using the SCORE for preventing serious CVD events	Adults 40–65 years old; without CVD, diabetes or CKD; specific risk factors may have been presented; no preventive pharmacotherapy	CVD risk assessment using the SCORE model	Standard care, without total risk assessment	(1) Cardiovascular death, (2) All-cause mortality, (3) Atherosclerotic major events.	Any discomfort reported by patients; a decrease in the quality of life; adverse physical, psychological or social outcomes	–
Karmali 2017 [39]	The effects of evaluating and providing CVD risk scores on CVD outcomes, risk factor levels, medication prescribing, and health behaviours	Adults (≥18 years) in outpatient settings free of clinical CVD. Patients with diabetes, elevated risk factors, preventive medications eligible	Systematic provision of a CVD risk score by a clinician, healthcare professional, or healthcare system	Usual care (i.e. no systematic provision of a CVD risk score)	Primary: CVD events; changes in total cholesterol, LDL cholesterol, systolic BP, diastolic BP, multivariable CVD risk. Secondary: preventive medication; medication adherence; smoking cessation; exercise; diet; decisional conflict; quality of life; costs.	Primary study investigator-defined adverse events e.g. physical or psychosocial events	overall low quality; 38 out of 41studies had high or unclear risk of bias
Collins 2017 [40]	Impact of global cardiovascular risk assessment in the primary prevention	Adults with no history of CVD	Interventions involving global CVD risk assessment	No formal risk assessment	Primary: CVD-related morbidity and mortality and all-cause mortality; Secondary: SBP, cholesterol level, quitting smoking	No	1 low, 6 fair, 3 medium, 2 medium to high, 1 high, 3 good quality studies

*BP* Blood pressure, *CVD* cardiovascular disease, *SR* systematic review, *RCT* randomised control trials, *CKD* chronic kidney disease, *SBP* systolic BP

The review performed by Brindle et al. [16] focused both on the accuracy and impact of CVD risk assessment in primary prevention. The authors performed comprehensive searches across 6 databases. The majority of included studies focused on CVD risk assessment accuracy. The authors identified 4 RCTs assessing effectiveness of using CVD risk score. As the quality of evidence was unclear, the authors concluded that it was insufficient to support the claim that CVD risk assessment performed by clinicians improves health outcomes.

Sheridan and Crespo [17] focused on the benefits and harms of physicians’ knowledge of CVD risk scoring. The authors searched one database (MEDLINE) and identified 11 primary studies. They concluded that “physicians’ knowledge of global CHD risk scores may translate into modestly increased prescribing of cardiovascular drugs and modest short-term reductions in CHD risk factors without clinical harm”.

The SR by Sheridan et al. [18] aimed at evaluating the effectiveness of presenting CVD risk information to patients as a primary intervention versus part of a multipart intervention. The researchers studied 4 databases and considered studies of any quantitative experimental design. They identified 18 primary studies of good or fair quality. Results suggested that providing CVD risk information to the patients may “improve accuracy of risk perception and may increase intent to initiate CHD prevention”. However, the effect on more long-term outcomes was not clear.

Waldron et al. [35] tried to assess the effectiveness of using different methods of communicating CVD risk and the impact of these methods on patient-related outcomes. The authors performed comprehensive searches across 6 databases. However, only 4 out of the 15 quantitative studies included assessed individuals’ actual risk. The majority of these studies were analogue studies using hypothetical risk profiles. The authors concluded that there were not many well-designed studies focusing on CVD risk communication.

In their review, Van Dieren et al. [36] aimed to identify all papers presenting CVD prediction models developed in either diabetic or general patient populations that included diabetes as a predictor. The review also identified studies examining the impact of applying a prediction model in clinical practice. They found 3 studies of this type, all of which utilised the Framingham prediction model. Results suggested that it is still unknown whether the use of these models indeed changes the treatment of patients with diabetes mellitus or whether it reduces the number of cardiovascular complications.

Willis et al. [19] aimed to evaluate the effectiveness of using validated CVD risk scores to identify individuals at highest risk followed by intervention as a means of reducing CVD risk or mortality. They included 5 full texts in their review. Two trials reported a reduction in CVD mortality. However one of them showed a lack of statistical significance, and in the second, the *p* value was not provided. The authors concluded that “evidence suggests that lifestyle interventions aimed at the primary prevention of CVD that use validated risk scores to recruit high risk individuals show potential for lowering CVD mortality and the incidence of cardiovascular events”.

Usher-Smith et al. [37] searched for interventional studies which involved providing a CVD risk assessment to patients or their providers. Final analysis was based on 17 primary studies. Results suggested that providing patients with risk information improves the accuracy of perceived risk without increasing anxiety or decreasing quality of life. Additionally, it leads to a small reduction in cholesterol and blood pressure despite a lack of evidence that this reduction is due to lifestyle changes. Moreover, providing risk information increases the frequency of prescribing lipid-lowering and blood pressure medications.

Tomasik et al. [38] focused only on the effect of using the specifically European total CVD risk estimation from SCORE. A comprehensive search did not reveal any study that compared important clinical outcomes (death, major events, adverse events) between groups that used the SCORE model and those who did not. The authors concluded that a demonstration of the benefits of using SCORE is still lacking and that current use of this tool as a preventive strategy is not supported by evidence.

The Cochrane review by Karmali et al. [39] focused on the effect of physicians, other healthcare professionals or the health system providing a multivariable CVD risk score. They identified 41 RCTs having multiple limitations, substantial heterogeneity and high or unclear risk of bias. Results suggested that the use of CVD risk scores has little or no effect on CVD events compared with standard care, though to a small extent it may reduce risk factor level (systolic BP, total cholesterol), multivariable CVD risk, or adverse events. It may also increase the use of antihypertensive and lipid-lowering pharmacotherapy.

The Collins et al. [40] review of systematic reviews summarises results from 6 SRs on the impact of global CVD risk assessment in primary prevention. No identified review reported results such as mortality or CVD morbidity. The authors also undertook ad hoc meta-analysis and extracted data from 16 primary studies of the identified publications. They showed small and possibly clinically insignificant reductions in systolic BP, cholesterol level and smoking cessation. The quality of evidence was low or very low (GRADE score).

The general conclusions about the included SRs is that there is currently little evidence that providing TRA has a significant impact on important patient outcomes.

### Biases in included systematic reviews and across systematic reviews

**Fig. 2:**
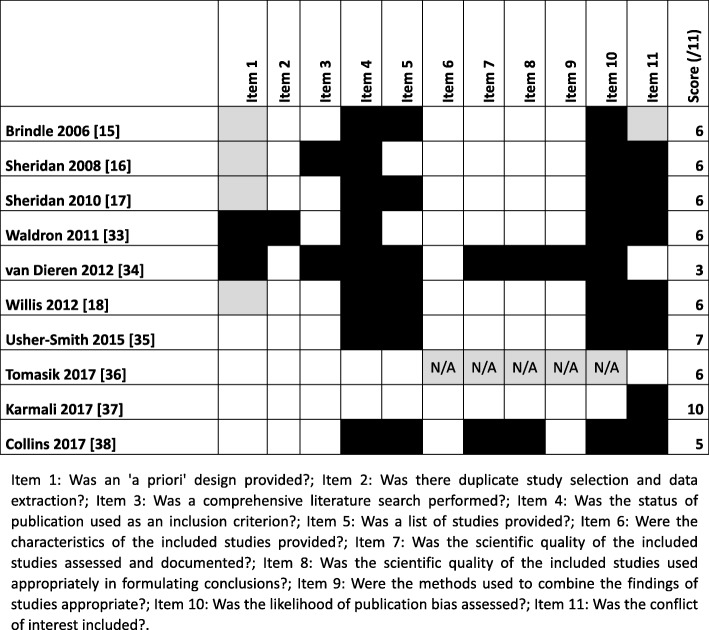
Assessment of the methodological quality of the included systematic reviews (AMSTAR). Legend: White = Yes; Black = No; Grey = can’t answer/unclear; N/A = not applicable

AMSTAR ratings ranged from 3/11 to 10/11 (mean 6.1/11); no SRs were excluded based on quality (see Fig. 2). Ten eligible SRs included 66 unique primary studies. Thirty five publications were encompassed in only one SR and 31 in more than one. The measure of overlap by corrected covered area (CCA) was 9%, indicating a moderate overlap [26]. Details of calculations and the list of primary studies are presented in Additional file 4.

In the majority of the SRs, the authors presented the same outcomes in the results sections that they had planned to assess in their initial protocols or in the methodology section of published reports. Collins et al. [40] deviated from their registered protocol, which they reported in Additional file 1, published online. They had originally planned to explore many different outcomes, but instead focused on what they deemed to be the most important ones: CVD-related morbidity and mortality as primary outcomes and systolic blood pressure, cholesterol, and smoking as secondary outcomes. Willis et al. [19] additionally reported cardiovascular disease risk factors which were not in the original scope of their work.

### Effects of interventions

#### Primary outcomes

##### CVD death and fatal and non-fatal CVD event

The SR by Willis et al. [19] included two original studies reporting changes in coronary [CHD] death [41, 42]. In one of the studies [41] the authors reported a lowering of CVD deaths of 4.7% without indicating if the difference is statistically significant. In another study [42], the reduction of CHD deaths of 7.4% was not statistically significant (*p* > 0.05). These two original studies were not mentioned or included in any other SR. Both studies were published in the 1980’s, when strategies for CVD risk reduction were far more limited. Additionally, these trials were really designed with the intent of examining the effectiveness of a high intensity intervention geared at risk factor modification, rather than the use of risk scores. Both studies only included men.

Karmali et al. [39] presented a meta-analysis of three studies on the effect of providing TRA on fatal and non-fatal CV events. It reported no effect compared with conventional care (5.4% vs 5.3%; risk ratio [RR] 1.01, 95% confidence interval [CI] 0.95 to 1.08; I^2^ = 25%). Included in meta-analysis studies had low quality with high or unclear risk of bias. Furthermore, 2 of these studies were likely underpowered for assessment of CVD events due to limited recruitments of participants over the age of 50 years and low rates of CVD events. The third study included a cohort of patients with HIV, which is not generalizable.

##### Adverse events

Authors of three SRs [17, 37, 39] were looking for possible adverse events of CVD risk assessment. These SRs showed no difference in the presence of psychological symptoms in groups with or without TRA. Usher-Smith et al. [37] also showed a small improvement in psychological well-being (measured with GHQ-28 scores) and level of anxiety in high CVD risk patients after TRA.

One SR [39] reported low-quality evidence suggesting that providing TRA may reduce the presence of adverse physical events (1.9% vs 2.7%; RR 0.72, 95% CI 0.49 to 1.04; I^2^ = 0%).

The detailed results of all primary outcomes are presented in Table 2.

**Table 2:** Effect of TRA on CVD deaths, CVD events, and adverse events reported in 10 SRs (primary outcomes). [*In case of meta-analysis (MA), the number of studies included and the results were presented; results of studies not included in the MA are shown below]*

SRs	CVD death	Fatal and non-fatal CVD event	Adverse events		
			*physical*	*psychological*	*social*
Brindle 2006 [16]	–	–	–	–	–
Sheridan 2008 [17]	–	–	–	Psychological symptoms after labelling: Mixed: 1 study; No difference: 4 studies	–
Sheridan 2010 [18]	–	–	–	–	–
Waldron 2011 [35]	–	–	–	–	–
van Dieren 2012 [36]	–	–	–	–	–
Willis 2012 [19]	Reduction: 1 study (CVD deaths −4,7%, statistical significance not reported) No difference: 1 study (CHD deaths −7,4%, p > 0.05)	–	–	–	–
Usher-Smith 2015 [37]	–	–	–	Psychological well-being: No difference: 3 studies Psychological well-being in high CVD risk group: Improvement: 1 study Anxiety: No difference: 2 studies Anxiety in high CVD risk group: Reduction: 1 study Worry about future risk of heart attacks: No difference:1 study	–
Tomasik 2017 [38]	–	–	–	–	–
Karmali 2017 [39]	–	No difference: MA, 3 studies (RR 1.01; 95% CI 0.95 to 1.08; I^2^ = 25%)	Adverse events defined by authors:^a^ No difference: MA, 4 studies (RR 0.72, 95% CI 0.49 to 1.04; I^2^ = 0%)	Anxiety: No difference: MA, 2 studies (SMD − 0,07, 95% CI -0,27 to 0,13; I^2^ = 0%); 1 study Psychological distress: No difference: 1 study	
			Health-related quality of life^b^: No difference: 1 study		
Collins 2017 [40]	–	–	–	–	–

^a^Anxiety inclusion unclear; ^b^Subjective evaluation of either physical or mental health

*MA* meta-analysis, *NS* non-significant, *CVD* cardiovascular disease, *CHD* coronary heart disease, *RR* risk ratio, *SMD* standardised mean differences

#### Secondary outcomes

##### All-cause mortality

None of the included SRs reported changes in all-cause mortality.

##### Change in predicted global CVD risk and CVD risk factors.

Four SRs reported reduction of predicted global CVD risk after providing TRA [17, 19, 37, 39]. One SR [18] showed mixed results and another SR [16] showed no difference. Seven SRs showed a tendency towards a small reduction of BP [16–19, 37, 39, 40]. The authors of eight SRs assessed possible changes in cholesterol level. They showed mixed results with the tendency towards reduction of TC [16–19, 35, 37, 39, 40]. There was no change in reported obesity level [17, 37], diet used [16, 37, 39] or alcohol consumption [37], but a slight reduction in smoking was noted [18, 19, 37, 39, 40].

Details are presented in Table 3.

**Table 3:** Effect of TRA on global CVD risk, single risk factors level, lifestyle, and other factors (secondary outcomes). *[In case of meta-analysis (MA), the number of studies included and the results were presented; results of studies not included in the MA are shown below]*

SR	Global CVD risk	CVD risk factors				Lifestyle				Other
		BP	TC	LDL-C	Obesity	Smoking	Exercise	Diet	Alcohol	
Brindle 2006 [16]	No difference: 1 study	Reduction: 1 study; No difference: 2 studies	No difference: 1 study	–	–	–	–	–	–	Referral to dietician: No difference: 1 study
Sheridan 2008 [17]	Reduction: 1 study	Reduction: 1 study; No difference: 1 study	Reduction: 1 study	Reduction: 1 study	No difference: 1 study	No difference: 2 studies	Increase: 1 study	–	–	Referral to dietician: No difference: 1 study
Sheridan 2010 [18]	Reduction: 4 studies; No difference: 5 studies	Reduction: 5 studies; No difference: 3 studies	Reduction: 3 studies; Increase: 1 study	Reduction: 2 studies; No difference: 1 study	–	Reduction: 1 studies; No difference: 5 studies	Increase: 1 study; Mixed: 4 studies	No difference: 3 studies	–	
Waldron 2011 [35]	–	–	Reduction: 1 study	–	–	–	–	–	–	–
van Dieren 2012 [36]	–	–	–	–	–	–	–	–	–	–
Willis 2012 [19]	Reduction: 1 study	Reduction: 1 study; No difference: 4 studies	Reduction: 3 studies; No difference: 2 studies	–	–	Reduction: 1 study	–	–	–	–
Usher-Smith 2015 [37]	Reduction: MA, 4 studies (−0,39 MD; 95% CI -0.71 to − 0.07; I^2^ = 62,9%), High CVD risk: No difference: 1 study	SBP: No difference: MA, 4 studies (− 0,82 mmHg; 95% CI -2.70 to 1.05; I^2^ = 27,4%) No difference: 1 study SBP, high risk: Reduction: MA, 2 studies (−4,82 mmHg; 95% CI -9.38 to − 0.26) DBP: No difference: MA, 3 studies (− 0,48 mmHg, 95% CI -1.41 to 0.44, I^2^ = 0%) DBP, high risk: No difference: 1 study (−1,9 mmHg, NS)	No difference: MA, 4 studies (− 0,11 mmol/l; 95% CI -0.23 to 0.01; I^2^ = 69,9%); No difference: 1 study; Reduction: 1 study		No difference: 2 studies	No difference: 4 studies	No difference: 2 studies	No difference: 3 studies	No difference: 2 studies	HDL-C and TC to HDL-C ratio: No difference: 1 study TG: No difference: 1 study Reach lipids targets: Increase: 1 study Glycaemia: No difference: 1 study Accurate risk perception: Increase: 3 studies; No difference: 2 studies Healthcare usage: Mixed: 2 studies
Tomasik 2017 [38]	–	–	–	–	–	–	–	–	–	–
Karmali 2017 [39]	Reduction: MA, 9 studies (slightly reduced SMD − 0,21; 95% CI -0.39 to − 0.02; I^2^ = 94%); No difference: 4 studies; Reduction: 1 study	SBP: Reduction: MA, 16 studies (MD − 2,77 mmHg; 95% CI -4.16 to − 1.38; I^2^ = 93%); No difference: 2 studies DBP: Reduction: MA, 14 studies (MD − 1,12 mmHg; 95% CI -2.11 to − 0.13; I^2^ = 94%); No difference: 2 studies	Reduction: MA, 12 studies (MD −0,10 mmol/l; 95% CI -0.20 to 0.00; I^2^ = 94%)	No diference: MA,10 studies (MD − 0,03 mmol/l; 95% CI -0.10 to 0.04; I^2^ = 84%)	–	Reduction: MA,7 studies (RR 1,38; 95% CI 1.13 to 1.69; I^2^ = 0%); Reduction: 5 studies; No difference: 4 studies	No difference: MA, 2 studies (RR 0,98; 95% CI 0.90 to 1.06; I^2^ = 0%); No difference: 3 studies; Increase: 3 studies	No difference: 4 studies; Increase: 2 studies	–	Decisional conflict: Reduction MA, 4 studies (SMD-0,29; 95% CI -0,57 to − 0,01; I^2^ = 79%) Costs: Reduction in cost of lipid-lowering medications prescribed to low-risk individuals: 1 study
Collins 2017 [40]	–	SBP: Reduction: MA, 9 studies (MD − 2,22 mmHg; 95% CI -3.49 to − 0.95; I^2^ = 66%); Reduction: 2 studies; No difference: 1 study	Reduction (MA, 5 studies (MD − 0,11 mmol/l; 95% CI -0.20 to − 0.02; I^2^ = 72%); Reduction: 2 studies	Reduction (MA, 4 studies (MD − 0,15 mmol/l; 95% CI -0.26 to − 0.05; I^2^ = 47%); No difference: 1 study	–	Reduction: MA,7 studies (1,62 RR of quitting; 95% CI 1.08 to 2.43; I^2^ = 17%); Reduction: 1 study; No difference: 1 study	–	–	–	–

*BP* blood pressure, *SBP* systolic blood pressure, *DBP* diastolic blood pressure, *TC* total cholesterol, *LDL-C* low-density lipoprotein, *HDL-C* high density lipoprotein, *MA* meta-analysis, *NS* non-significant, *RR* risk ratio, *CI* confidence interval, *MD* mean difference

##### Prescription of risk-reducing drugs according and against guidelines

Five SRs [16, 17, 36, 37, 39] reported the impact of TRA on prescription of risk-reducing drugs. The results were mixed, but in all they suggest that TRA use may increase or have no effect on prescription of aspirin, anti-hypertensive and lipid-lowering drugs in all CVD risk groups. They also suggest that prescription of anti-hypertensive and lipid-lowering drugs in high CVD risk groups is increased.

One SR [39] reported the effect of providing TRA to patients on prescription of drugs against guidelines.

Detailed data is presented in Table 4.

**Table 4:** Effect of TRA on drugs prescription in relation to guidelines (secondary outcomes)

SR	According to guidelines				Against guidelines
	Aspirin	Antihypertensive	lipid-lowering	antidiabetic	
Brindle 2006 [16]		All CVD risk groups: No difference: 1 study High CVD risk group: Increase: 1 study	All CVD risk groups: No difference: 1 study High CVD risk group: Increase: 1 study	No difference: 1 study	–
Sheridan 2008 [17]		All CVD risk groups: No difference: 1 study High CVD risk group: Increase: 1 study	All CVD risk groups: No difference: 2 studies High CVD risk group: Increase: 1 study; No difference: 1 study	All CVD risk groups: No difference: 1 study High CVD risk group: No difference: 1 study	–
Sheridan 2010 [18]	–	–	No difference: 1	–	–
Waldron 2011 [35]	–	–	–	–	–
van Dieren 2012 [36]	–	Increase: 2 studies; No difference: 1 study	Increase: 2 studies; No difference: 1 study	No difference: 1 study	–
Willis 2012 [19]	–	–	–	–	–
Usher-Smith 2015 [37]	–	All CVD risk groups: No difference: 3 studies; High CVD risk group: Increase: 1 study	All CVD risk groups: No difference: MA, 4 studies (RR 1,35, 95% CI 0.96 to 1.90, I^2^ = 0%) High CVD risk group: Increase: MA, 3 studies (RR 1,83, 95% CI 1.13 to 2.98)	All CVD risk groups: No difference: 3 studies; High CVD risk group: No difference: 2 studies	–
Tomasik 2017 [38]	–	–	–		–
Karmali 2017 [39]	Increase: MA, 3 studies (RR 2,71; 95% CI 1.24 to 5.91; I^2^ = 0%); No difference: 2 studies	Increase: MA, 8 studies (RR 1,51, 95% CI 1.08 to 2.11, I^2^ = 53%); No difference: 3 studies	Increase: MA, 11 studies (RR 1,47, 95% CI 1.15 to 1.87, I^2^ = 40%); No difference: 4 studies		Low CVD risk group: Reduction: 1 study
Collins 2017 [40]	–	–	–	–	–

*MA* meta-analysis, *NS* non-significant, *RR* risk ratio, *CI* confidence interval, *CVD* cardiovascular disease

We were unable to find any studies comparing the use of different risk models followed by structured or unstructured intervention showing that one is more effective than another.

The majority of SRs (*n* = 8) recommend that further effectiveness studies are needed. Five SRs propose high quality RCTs. One of these SRs proposes a cluster RCT with CVD mortality and morbidity data as primary outcomes. Two SRs propose trials with surrogate outcomes (CVD risk factor levels), which may be more feasible due to a smaller population and less time needed.

## Discussion

### Main findings

We have identified and analysed 10 SRs which were published between 2006 and 2017. Two of them reported the impact of performing TRA on mortality, fatal and non-fatal CV events. One medium quality SR [19] revealed 2 primary studies reporting reduction of CVD death, but it was statistically insignificant or significance was not reported in the original study. Other high quality SR [39] presented meta-analysis of 3 primary studies which showed no significant differences concerning fatal and non-fatal CV events. Primary studies included in both SRs had important limitations.

Three SRs [17, 37, 39] have analysed the impact of performing TRA on adverse events. It was shown that using TRA causes no difference in presence of psychological or physical events.

The impact of TRA on global CVD risk and individual risk factors is ambiguous, but a tendency towards slight reduction of BP, TC and smoking levels, especially in high risk patients group, was observed. TRA had no influence on lifestyle behaviour.

### Strengths and limitations

To the best of our knowledge this is the largest overview of SRs in this area. We included 10 SRs while a previous overview published in March 2017 covered six [40]. Our overview of SRs was conducted strictly according to the protocol, which was registered with the PROSPERO database, and later published in the BMJ Open [20]. Other strengths are a robust search strategy prepared by an experienced librarian, and broad inclusion criteria.

There are some obvious limitations to our overview of SRs. We did not review the quality of each study included in the SRs, nor perform an independent analysis of data (which is standard procedure when performing overview of SRs). The electronic search strategy was limited to 4 main international bibliographic databases and English language literature only. Grey literature for unpublished papers was not studied, however a search for ongoing or recently completed systematic reviews in the PROSPERO database was performed. In addition, we also manually reviewed the reference lists of all included SRs.

### Comparison with other studies and opinions

Collins et al. [40] completed searches for their overview of SR in October 2016. They did not find SRs concerning their primary outcomes (CVD mortality and morbidity and all-cause mortality). The conclusions of that overview differ from ours and the authors stated that “there is currently no evidence that the prospective use of global cardiovascular risk assessment translates to reductions in CVD morbidity or mortality”. We hold the view that there is a limited evidence for the lack of these reductions.

Results of our overview of SRs can be also compared with evidence constituting the basis for recommendations within national and international guidelines. Major clinical practice guidelines (CCS [13], ESC/ESH [6], ACC/AHA [14], JBS3 [15], NICE [43]) recommend assessing risk of CVD using TRA. It is stated that use of these risk scores increases the accuracy of prediction of CVD events and guides management decisions in primary prevention. Some guidelines indicate that there is no proof for the effectiveness of TRA [6]. Our research has shown the contrary - there is a limited evidence, of low overall quality, for a lack of effectiveness of TRA on CVD events.

There are publications indicating, similar to our study, that some primary prevention interventions may not be effective. The Cochrane review for instance concluded that general health checks performed in healthy individuals do not reduce morbidity or mortality, nor in overall cardiovascular or cancer causes. [32] The Danish Inter99 randomised trial revealed that systematic screening for risk factors of ischaemic heart disease, followed by individually tailored intervention had no effect after 10 years and did not reduce incidence of stroke, ischaemic heart disease, as well as mortality rate [44]. On the other hand Si S. et al. showed that health checks are associated with statistically significant, though clinically small, improvements in surrogate outcomes, especially in high risk individuals [33].

### Interpretation of results

The readers of our overview should take into consideration that the results concerning the effectiveness of TRA comes not from primary studies but are strictly limited to results published in SRs and meta-analyses. Data derived from these types of studies are the most useful evidence in clinical practice [6]. However, our analysis shows a diversity in the quality of included SRs. The majority of them are, according to AMSTAR, of medium quality. We also present the quality of evidence of primary studies as assessed by authors of the included SRs. Most of them reported a low quality of primary studies. In accordance with the authors of these SRs, we agree that conducting further research is still necessary, in order to obtain more persuasive evidence for TRA effectiveness in primary prevention.

There are many reasons why TRA may not be effective and they are described in the literature. Firstly, false positive results of TRA may cause medical treatment which is unnecessary and brings risk of side effects. Secondly, false negative results may cause the lack of appropriate treatment [6]. Thirdly, only a small number of risk variables are included in most of the models (they do not include e.g. family history, TG level, or obesity). Risk categories can be arbitrary (e.g. low, medium, high). In addition, mortality and morbidity rates are declining in most European countries but prediction models are based on historic data and may not apply to the population to be assessed. Moreover, a small change in one of the individual risk factors (e.g. blood pressure) from 1 day to another can markedly change patients’ estimated CVD risk. Finally many physicians identify their patients’ CVD risk factors and launch appropriate treatment without the necessity of TRA [45].

### Implications

Even though TRA is recommended by major clinical practice guidelines, medical professionals should be aware that current, limited and of low quality, evidence from SRs indicate a possible lack of its clinical effectiveness. Using TRA tools will not harm the patients. There are no grounds to conclude that, in clinical practice, CVD risk assessment based on other than TRA methods, e.g. only on one particular risk factor or counting the number of risk factors instead of using TRA, is inappropriate. However, there are several other potential benefits associated with the use of TRA which are considered in the current guidelines.

Guideline authors should consider re-evaluating the class of recommendations. In the 2016 European Guidelines on CVD prevention [6], the recommendation for routine use of the SCORE algorithm in primary prevention of CVD is marked as class I. This means that there is “evidence and/or general agreement that a procedure is beneficial, useful, effective”. Results of our overview indicate that in this case, class IIb (“usefulness/efficacy is less well established by evidence/opinion”) would be more appropriate. In the British NICE clinical guideline CG181 [43] (concerning lipid modification for the primary and secondary prevention of CVD), the strength of the recommendations in individuals without CVD should also be lowered from “offer” to “consider”.

Researchers should plan and perform impact studies (probably RCTs) which take into account important endpoints e.g. CVD mortality and morbidity, to ultimately confirm the effectiveness or lack of effectiveness of TRA. Development of a new model of TRA and looking for the most appropriate format of its communication also seems inappropriate when effectiveness is not proven. Due to the probability of TRA ineffectiveness, the search for an utterly new method allowing the indication of points of onset and intensification of intervention in CVD primary prevention should be started.

Both researchers and guideline authors should be aware that it is inappropriate to use the models prepared and validated for prediction purposes to make clinical decisions concerning patients. It may be especially misleading if thresholds are based on opinions (which are cheap and quick) and not on impact studies.

Founders and policymakers ought to be aware that spending resources on adopting TRA into practice may be against current evidence which is not strong.

In the protocol of our overview, we expressed optimism that our study might allow patients to have information about a strong scientific background of performed CVD risk assessment. We had hoped that this knowledge would boost patients’ adherence to their physicians’ recommendations. However, these hopes have turned out to have been in vain.

## Conclusion

SRs identified and included in our overview show a limited evidence, of low overall quality, suggesting a possible lack of effectiveness of TRA in reducing CVD events and mortality, as well as a clinically insignificant influence on individual risk factor levels. These studies also reveal that using TRA does not cause harm to patients. Presented results cannot be considered final and further research is still necessary, however broad implementation of TRA by practice might be devoid of justification based on evidence and should be reconsidered.

## Additional files

Additional file 1: Deviations from the protocol. (DOCX 12 kb)

Additional file 2: MEDLINE search strategy. (DOCX 19 kb)

Additional file 3: PRISMA checklist. (DOCX 26 kb)

Additional file 4: the measure of overlap by corrected covered area. (DOCX 32 kb)

## Abbreviations

ACC/AHA: American College of Cardiology/American Heart Association
BP: Blood pressure
CCA: Corrected cover area
CI: Confidence interval
CVD: Cardiovascular disease
DBP: Diastolic blood pressure
HDL-C: High density lipoprotein
LDL-C: Low-density lipoprotein
MA: Meta-analysis
MD: Mean difference
NS: Non-significant
RCTs: Randomised control trials
RR: Risk ratio
SBP: Systolic blood pressure
SCORE: Systematic COronary Risk Evaluation
SRs: Systematic reviews
TC: Total cholesterol
TRA: Total risk assessment
WHO: World Health Organization
