# Supplementary material for: Effect of using cardiovascular risk scoring in routine risk assessment in primary prevention of cardiovascular disease: an overview of systematic reviews
Source: BMC Cardiovasc Disord. 2019 Jan 9;19:11. doi: 10.1186/s12872-018-0990-2 (PMC6327540; doi:10.1186/s12872-018-0990-2)
Supplement: Supplementary file 1 — Deviations from the protocol. (DOCX 12 kb) [file 12872_2018_990_MOESM1_ESM.docx]

# **Appendix 1: Deviations from protocol**

Statement in the protocol; section Methods/Design; subsection Eligibility criteria: “Only studies performed in a primary care setting will be eligible.”

Since not all authors of the identified SRs specified exact setting of primary studies included in their reviews, we decided to extend inclusion criteria for outpatient setting.
