# Supplementary material for: Effect of using cardiovascular risk scoring in routine risk assessment in primary prevention of cardiovascular disease: an overview of systematic reviews
Source: BMC Cardiovasc Disord. 2019 Jan 9;19:11. doi: 10.1186/s12872-018-0990-2 (PMC6327540; doi:10.1186/s12872-018-0990-2)
Supplement: Supplementary file 2 — MEDLINE search strategy. (DOCX 19 kb) [file 12872_2018_990_MOESM2_ESM.docx]

# **Appendix 2: Search Strategy MEDLINE (Ovid)**

1. exp Cardiovascular Diseases/pc [Prevention & Control] (168685)
2. exp Hyperlipidemias/ (60259)
3. exp Blood Pressure/ (265572)
4. cardiovascular disease.mp. (94094)
5. cv.mp. (40614)
6. cvd.mp. (23109)
7. chd.mp. (19894)
8. vascular.mp. (649889)
9. coronary.mp. (433965)
10. heart.mp. (1075606)
11. cardio.mp. (14425)
12. arteriosclerosis.mp. (69727)
13. atherosclerosis.mp. (103374)
14. hypertension.mp. (410548)
15. blood pressure.mp. (391513)
16. hyperlipidemia.mp. (17927)
17. cholesterol.mp. (247234)
18. 1 or 2 or 3 or 4 or 5 or 6 or 7 or 8 or 9 or 10 or 11 or 12 or 13 or 14 or 15 or 16 or 17 (2607914)
19. prevention.mp. (466325)
20. "primary prevention".mp. (27061)
21. "primary intervention".mp. (712)
22. "health promotion".mp. (72921)
23. "health education".mp. (76465)
24. "life style".mp. (54902)
25. "risk control".mp. (465)
26. "mass screening".mp. (90950)
27. counseling.mp. (80338)
28. Health Education/ (55847)
29. Health Promotion/ (61356)
30. Life Style/ (48485)
31. Mass Screening/ (89012)
32. Counseling/ (31160)
33. Primary Prevention/ (15764)
34. 19 or 20 or 21 or 22 or 23 or 24 or 25 or 26 or 27 or 28 or 29 or 30 or 31 or 32 or 33 (769892)
35. 18 and 34 (120618)
36. risk factors/ (652618)
37. Risk Assessment/ (204497)
38. risk factor$.mp. (864725)
39. age.mp. (7488032)
40. smoking.mp. (231433)
41. cholesterol.mp. (247234)
42. HDL-C.mp. (12723)
43. aspirin.mp. (58634)
44. men.mp. (410062)
45. women.mp. (778645)
46. "multiple risk factors".mp. (2932)
47. Diabetes Mellitus/ (102397)
48. diabetes.mp. (482454)
49. Obesity/ (146314)
50. obesity.mp. (238130)
51. Hyperglycemia/ (23313)
52. Hyperglycemia.mp. (45379)
53. 36 or 37 or 38 or 39 or 40 or 41 or 42 or 43 or 44 or 45 or 46 or 47 or 48 or 49 or 50 or 51 or 52 (8549717)
54. 34 and 53 (455890)
55. 35 or 54 (488901)
56. Risk Assessment/ (204497)
57. "risk factors assessment".mp. (94)
58. "risk factor function".mp. (1)
59. prediction.mp. (167913)
60. prognosis.mp. (574395)
61. Prognosis/ (410580)
62. Forecast.mp. (5654)
63. Forecasting/ (75950)
64. equation.mp. (85887)
65. appraisal.mp. (27915)
66. calculation.mp. (74773)
67. estimate.mp. (266996)
68. model.mp. (1546840)
69. chart.mp. (44959)
70. engine.mp. (8709)
71. table.mp. (44842)
72. "scoring model".mp. (305)
73. instrument.mp. (90730)
74. calculator.mp. (2433)
75. Algorithms/ (203480)
76. algorithm$.mp. (290914)
77. "decision support system".mp. (2292)
78. "treatment decisions".mp. (9932)
79. 56 or 57 or 58 or 59 or 60 or 61 or 62 or 63 or 64 or 65 or 66 or 67 or 68 or 69 or 70 or 71 or 72 or 73 or 74 or 75 or 76 or 77 or 78 (3064830)
80. ARIC diabetes.tw. (3)
81. ARIC stroke.tw. (0)
82. ARIC.tw. (1182)
83. Albany.tw. (603)
84. Augsburg MONICA.tw. (12)
85. Bombay.tw. (1291)
86. British Regional Heart Study.tw. (121)
87. Cardiff Type 2 diabetes coronary heart disease risk function.tw. (0)
88. Chicago Heart Association Detection Project in Industry.tw. (55)
89. Chicago Western Electric.tw. (26)
90. Carta del Rischio del PPP.tw. (0)
91. Copenhagen stroke.tw. (49)
92. CUORE.tw. (152)
93. DECODE.tw. (1739)
94. Dubbo.tw. (121)
95. ERICA.tw. (327)
96. Family heart study.tw. (316)
97. FINE 2002.tw. (0)
98. FINRISK.tw. (153)
99. Framingham.tw. (7290)
100. Global coronary risk score.tw. (1)
101. Glostrup.tw. (322)
102. GREAT.tw. (231780)
103. GRIPS.tw. (851)
104. Gubbio.tw. (57)
105. (Hypertension Detection and Follow-up Programme).tw. (3)
106. INSIGHT.tw. (139721)
107. INDANA.tw. (17)
108. International Cooperative Study on Cardiovascular Epidemiology.tw. (1)
109. Italian cohorts of Seven countries study.tw. (1)
110. JBS3 risk score.tw. (0)
111. John Hopkins multiple risk equations.tw. (0)
112. Kaiser Permanente.tw. (2326)
113. Luoto.tw. (2)
114. Leoncini.tw. (6)
115. MESA Risk Score.tw. (3)
116. McManus.tw. (110)
117. "MRFIT CHD prevention model".tw. (0)
118. Nagpur.tw. (315)
119. New Zealand Cardiovascular Risk Charts.tw. (1)
120. North Karelia.tw. (338)
121. Northwick Park.tw. (305)
122. NHANES 1.tw. (18)
123. NHANES 2.tw. (2)
124. NHEFS.tw. (82)
125. North Sweden MONICA.tw. (0)
126. Olmsted County.tw. (1525)
127. Paris Prospective Study.tw. (79)
128. Plovdiv.tw. (316)
129. PRECARD.tw. (5)
130. PROCAM Assmann.tw. (0)
131. PROCAM Voss.tw. (0)
132. Il Progetto Romano di Prevenzione della Cardiopatia Coronarica.tw. (0)
133. QRISK.tw. (39)
134. QRISK2.tw. (47)
135. Rasmusen Centre.tw. (0)
136. RCV-ap.tw. (0)
137. REGICOR.tw. (119)
138. RIFLE.tw. (1457)
139. RISCARD.tw. (2)
140. Rotterdam Nijhuis.tw. (0)
141. Royal College of Physicians of Edinburgh Diabetes research group.tw. (0)
142. Sante Quebec Heart Health Survey.tw. (0)
143. Physicians Health Study.tw. (509)
144. Seven Countries.tw. (1182)
145. Tecumseh.tw. (255)
146. TEKHARF.tw. (6)
147. UKPDS.tw. (565)
148. US Railway.tw. (0)
149. West Dorset.tw. (8)
150. Western Collaborative group.tw. (51)
151. Westlund score.tw. (0)
152. WHO 1974.tw. (4)
153. Zoetermeer.tw. (33)
154. global.tw. (248719)
155. total.tw. (1589663)
156. absolute.tw. (132148)
157. ATP III CHD risk score.tw. (0)
158. "2013 ACC/AHA pooled cohort risk".tw. (2)
159. "American College of Cardiology/American Heart Association Pooled Cohort".tw. (4)
160. "RiskACC/AHA".tw. (2)
161. CERT.tw. (186)
162. Systematic Coronary Risk Evaluation.tw. (221)
163. SCORE.tw. (364402)
164. 80 or 81 or 82 or 83 or 84 or 85 or 86 or 87 or 88 or 89 or 90 or 91 or 92 or 93 or 94 or 95 or 96 or 97 or 98 or 99 or 100 or 101 or 102 or 103 or 104 or 105 or 106 or 107 or 108 or 109 or 110 or 111 or 112 or 113 or 114 or 115 or 116 or 117 or 118 or 119 or 120 or 121 or 122 or 123 or 124 or 125 or 126 or 127 or 128 or 129 or 130 or 131 or 132 or 133 or 134 or 135 or 136 or 137 or 138 or 139 or 140 or 141 or 142 or 143 or 144 or 145 or 146 or 147 or 148 or 149 or 150 or 151 or 152 or 153 or 154 or 155 or 156 or 157 or 158 or 159 or 160 or 161 or 162 or 163 (2516421)
165. 79 and 164 (530865)
166. 55 and 165 (22280)
167. (systematic$ adj review$).tw. (86652)
168. "Review"/ (2150016)
169. Meta-Analysis/ (70868)
170. (meta-anal$ or meta?anal$).tw. (96148)
171. systematic literature review$.tw. (5813)
172. 167 or 168 or 169 or 170 or 171 (2225199)
173. 166 and 172 (3133)
174. limit 173 to (english language and humans and yr="1990 -Current" and "all adult (19 plus years)") (799)

***************************

[mp=title, abstract, original title, name of substance word, subject heading word, keyword heading word, protocol supplementary concept word, rare disease supplementary concept word, unique identifier]
