# Supplementary material for: Effect of using cardiovascular risk scoring in routine risk assessment in primary prevention of cardiovascular disease: an overview of systematic reviews
Source: BMC Cardiovasc Disord. 2019 Jan 9;19:11. doi: 10.1186/s12872-018-0990-2 (PMC6327540; doi:10.1186/s12872-018-0990-2)
Supplement: Supplementary file 4 — the measure of overlap by corrected covered area. (DOCX 32 kb) [file 12872_2018_990_MOESM4_ESM.docx]

# **Appendix 4: The measure of overlap by corrected covered area (CCA)**

| **Systematic review**  **Study** | **Brindle 2006** | **Sheridan 2008** | **Sheridan 2010** | **Waldron 2011** | **van Dieren 2012** | **Willis 2012** | **Usher-Smith 2015** | **Tomasik 2017** | **Karmali 2017** | **Collins 2017** |
| --- | --- | --- | --- | --- | --- | --- | --- | --- | --- | --- |
| Hall et al. 2003 [1] | **+** | **+** |  |  | **+** |  | **+** |  | **+** |  |
| Montgomery et al. 2000 [2] | **+** | **+** |  |  | **+** |  |  |  | **+** | **+** |
| Hanon et al. 2000 [3] | **+** |  |  |  |  |  | **+** |  | **+** | **+** |
| Hetlevik et al. 1999 [4] | **+** |  |  |  | **+** |  |  |  | **+** |  |
| Jacobson et al. 2006 [5] |  | **+** |  |  |  |  |  |  | **+** |  |
| Lowensteyn et al. 1998 [6] |  | **+** |  |  |  |  | **+** |  | **+** | **+** |
| Ramachandran et al. 2000 [7] |  | **+** |  |  |  |  |  |  |  |  |
| van Steenkiste et al. 2007 [8] |  | **+** |  |  |  |  |  |  | **+** | **+** |
| Christensen et al. 1995 [9] |  | **+** |  |  |  |  | **+** |  |  |  |
| Christensen et al. 2004 [10] |  | **+** |  |  |  |  | **+** |  | **+** |  |
| Connelly et al. 1998 [11] |  | **+** |  |  |  |  | **+** |  |  |  |
| Marteau et al. 1996 [12] |  | **+** |  |  |  |  |  |  |  |  |
| Meland et al. 1996 [13] |  | **+** |  |  |  |  |  |  |  |  |
| Avis et al. 1989 [14] |  |  | **+** |  |  |  | **+** |  |  |  |
| Christian et al. 2005 [15] |  |  | **+** |  |  |  |  |  |  |  |
| Grover et al. 2007 [16] |  |  | **+** | **+** |  |  | **+** |  | **+** | **+** |
| Grover et al. 2009 [17] |  |  | **+** |  |  |  | **+** |  |  |  |
| Hanlon et al. 1995 [18] |  |  | **+** |  |  |  | **+** |  | **+** | **+** |
| Paterson et al. 2002 [19] |  |  | **+** |  |  |  | **+** |  |  |  |
| Sheridan et al. 2006 [20] |  |  | **+** |  |  |  |  |  | **+** |  |
| Weymiller et al. 2007 [21] |  |  | **+** |  |  |  |  |  |  |  |
| Edelman et al. 2006 [22] |  |  | **+** |  |  |  |  |  | **+** |  |
| Krones et al. 2008 [23] |  |  | **+** |  |  |  |  |  | **+** |  |
| Lalonde et al. 2004 [24] |  |  | **+** |  |  |  |  |  |  |  |
| Lovibond et al. 1986 [25] |  |  | **+** |  |  |  |  |  |  | **+** |
| OXCHECK Study Group. 1994 [26] |  |  | **+** |  |  |  |  |  |  | **+** |
| OXCHECK Study Group. 1995 [27] |  |  | **+** |  |  |  |  |  |  | **+** |
| Turnbull et al. 2006 [28] |  |  | **+** |  |  |  |  |  |  | **+** |
| Williams et al. 2006 [29] |  |  | **+** |  |  |  |  |  | **+** | **+** |
| Family Heart Study Group. 1994 [30] |  |  | **+** |  |  |  |  |  | **+** | **+** |
| Emmett et al. 2005 [31] |  |  | **+** |  |  |  |  |  |  | **+** |
| Montgomery et al. 2003 [32] |  |  | **+** |  |  |  |  |  | **+** |  |
| Wister et al. 2007 [33] |  |  | **+** |  |  | **+** |  |  | **+** | **+** |
| Asimakopoulou et al. 2008 [34] |  |  |  | **+** |  |  | **+** |  |  |  |
| Charlson et al. 2008 [35] |  |  |  | **+** |  |  |  |  |  |  |
| Straus 2002 [36] |  |  |  | **+** |  |  |  |  |  |  |
| Meland et al. 1997 [37] |  |  |  |  |  | **+** |  |  |  |  |
| Ketola et al. 2001 [38] |  |  |  |  |  | **+** |  |  |  |  |
| MRFIT Research Group. 1982 [39] |  |  |  |  |  | **+** |  |  |  |  |
| Kornitzer et al. 1985 [40] |  |  |  |  |  | **+** |  |  |  |  |
| Hussein et al. 2008 [41] |  |  |  |  |  |  | **+** |  |  |  |
| Persell et al. 2013 [42] |  |  |  |  |  |  | **+** |  |  |  |
| Price et al. 2011 [43] |  |  |  |  |  |  | **+** |  |  | **+** |
| Qureshi et al. 2012 [44] |  |  |  |  |  |  | **+** |  |  |  |
| Bucher et al. 2010 [45] |  |  |  |  |  |  | **+** |  | **+** | **+** |
| Benner et al. 2008 [46] |  |  |  |  |  |  |  |  | **+** |  |
| Bertoni et al. 2009 [47] |  |  |  |  |  |  |  |  | **+** |  |
| Cobos et al. 2005 [48] |  |  |  |  |  |  |  |  | **+** |  |
| Denig et al. 2014 [49] |  |  |  |  |  |  |  |  | **+** |  |
| Eaton et al. 2011 [50] |  |  |  |  |  |  |  |  | **+** |  |
| Engberg et al. 2002 [51] |  |  |  |  |  |  |  |  | **+** |  |
| Holt et al. 2010 [52] |  |  |  |  |  |  |  |  | **+** |  |
| Jorgensen et al. 2014 [53] |  |  |  |  |  |  |  |  | **+** |  |
| Koelewijn-van Loon et al. 2010 [54] |  |  |  |  |  |  |  |  | **+** |  |
| Lopez-Gonzalez et al. 2015 [55] |  |  |  |  |  |  |  |  | **+** |  |
| Mann et al. 2010 [56] |  |  |  |  |  |  |  |  | **+** |  |
| Peiris et al. 2015 [57] |  |  |  |  |  |  |  |  | **+** |  |
| Perestelo-Perez 2016 [58] |  |  |  |  |  |  |  |  | **+** |  |
| Persell et al. 2015 [59] |  |  |  |  |  |  |  |  | **+** | **+** |
| Sheridan et al. 2011 [60] |  |  |  |  |  |  |  |  | **+** |  |
| Soureti et al. 2011 [61] |  |  |  |  |  |  |  |  | **+** |  |
| Turner et al. 2012 [62] |  |  |  |  |  |  |  |  | **+** |  |
| Vagholkar et al. 2014 [63] |  |  |  |  |  |  |  |  | **+** |  |
| Webster et al. 2010 [64] |  |  |  |  |  |  |  |  | **+** |  |
| Welschen et al. 2012 [65] |  |  |  |  |  |  |  |  | **+** |  |
| Zullig et al. 2014 [66] |  |  |  |  |  |  |  |  | **+** |  |

Citation matrix:

- 66 primary publications (rows)
- 10 reviews (columns)

Frequency of encompassment of primary publications in 10 SRs:

|  | Number | Cumulative number | Percentage | Cumulative percentage |
| --- | --- | --- | --- | --- |
| 1 | 35 | 35 | 53,03030 | 53,0303 |
| 2 | 18 | 53 | 27,27273 | 80,3030 |
| 3 | 6 | 59 | 9,09091 | 89,3939 |
| 4 | 4 | 63 | 6,06061 | 95,4545 |
| 5 | 3 | 66 | 4,54545 | 100,0000 |
| absences | 0 | 66 | 0,00000 | 100,0000 |

35 publications were encompassed in only one SR and 31 in more than one:

- 18 publications were encompassed in 2 SRs
- 6 publications were encompassed in 3 SRs
- 4 publications were encompassed in 4 SRs
- 3 publications were encompassed in 5 SRs

**% overlap** = 31 / 66 = 0,4(69) ≈ 0,47 = 47%

**CA** = (4+11+20+4+3+5+17+0+39+17) / (66 × 10) = 120/660 = 0,(18) = 0,18 = 18%

**CCA** = (120-66) / (660-66) = 54 / 594 = 0,(09) = 0,09 = 9%

**According to Pieper et al.[67] CCA 9% can be considered as a moderate overlap.**

Abbreviations: CA - covered area; CCA - corrected covered area.

**References:**

1 Hall LML, Jung RT, Leese GP. Controlled trial of effect of documented cardiovascular risk scores on prescribing. *BMJ* 2003;**326**:251–2.http://www.pubmedcentral.nih.gov/articlerender.fcgi?artid=140760&tool=pmcentrez&rendertype=abstract

2 Montgomery AA, Fahey T, Peters TJ, *et al.* Evaluation of computer based clinical decision support system and risk chart for management of hypertension in primary care: randomised controlled trial. *Bmj* 2000;**320**:686–90. doi:10.1136/bmj.320.7236.686

3 Hanon O, Franconi G, Mourad JJ, *et al.* [The estimation of cardiovascular risk in hypertensive patients is not modified by management of the hypertension]. *Arch Mal Coeur Vaiss* 2000;**93**:943–7.http://www.ncbi.nlm.nih.gov/pubmed/10989734

4 Hetlevik I, Holmen J, Kruger O. Implementing clinical guidelines in the treatment of hypertension in general practice. Evaluation of patient outcome related to implementation of a computer-based clinical decision support system. *Scand J Prim Health Care* 1999;**17**:35–40. doi:10.1017/S0266462300161185

5 Jacobson T a, Gutkin SW, Harper CR. Effects of a global risk educational tool on primary coronary prevention: the Atherosclerosis Assessment Via Total Risk (AVIATOR) study. *Curr Med Res Opin* 2006;**22**:1065–73. doi:10.1185/030079906X104605

6 Lowensteyn I, Joseph L, Levinton C, *et al.* Can computerized risk profiles help patients improve their coronary risk? The results of the Coronary Health Assessment Study (CHAS). *Prev Med (Baltim)* 1998;**27**:730–7. doi:10.1006/pmed.1998.0351

7 Ramachandran S, Labib MH. Hyperlipidaemia and primary prevention of coronary heart disease: are the right patients being treated? *J Cardiovasc Risk* 2000;**7**:245–9.http://www.ncbi.nlm.nih.gov/pubmed/11006894

8 Steenkiste B, Weijden T, Stoffers HE, *et al.* Improving cardiovascular risk management: a randomized, controlled trial on the effect of a decision support tool for patients and physicians. *Eur J Cardiovasc Prev Rehabil* 2007;**14**:44–50. doi:10.1097/01.hjr.0000239475.71805.1e

9 Christensen B. Psychological reactions to information about risk of ischaemic heart disease in general practice. *Scand J Prim Health Care* 1995;**13**:164–7. doi:10.3109/02813439508996756

10 Christensen B, Engberg M, Lauritzen T. No long-term psychological reaction to information about increased risk of coronary heart disease in general practice. *Eur J Cardiovasc Prev Rehabil* 2004;**11**:239–43. doi:10.1097/01.hjr.0000129739.30593.23

11 Connelly J, Cooper J, Mann A, *et al.* The psychological impact of screening for risk of coronary heart disease in primary care settings. *J Cardiovasc Risk* 1998;**5**:185–91. doi:10.1177/174182679800500308

12 Marteau TM, Kinmonth a L, Thompson S, *et al.* The psychological impact of cardiovascular screening and intervention in primary care: a problem of false reassurance? British Family Heart Study Group. *Br J Gen Pract* 1996;**46**:577–82.

13 Meland E, Laerum E, Maeland JG. Life style intervention in general practice: effects on psychological well-being and patient satisfaction. *Qual Life Res* 1996;**5**:348–54.http://www.ncbi.nlm.nih.gov/pubmed/8763803

14 Avis NE, Smith KW, McKinlay JB. Accuracy of perceptions of heart attack risk: What influences perceptions and can they be changed? *Am J Public Health* 1989;**79**:1608–12. doi:10.2105/AJPH.79.12.1608

15 Christian AH, Mochari HY, Mosca LJ. Coronary Heart Disease in Ethnically Diverse Women: Risk Perception and Communication. Mayo Clin. Proc. 2005;**80**:1593–9. doi:10.4065/80.12.1593

16 Grover SA, Lowensteyn I, Joseph L, *et al.* Patient knowledge of coronary risk profile improves the effectiveness of dyslipidemia therapy: the CHECK-UP study: a randomized controlled trial. *Arch Intern Med* 2007;**167**:2296–303. doi:10.1001/archinte.167.21.2296

17 Grover SA, Lowensteyn I, Joseph L, *et al.* Discussing coronary risk with patients to improve blood pressure treatment: Secondary results from the CHECK-UP study. *J Gen Intern Med* 2009;**24**:33–9. doi:10.1007/s11606-008-0825-4

18 Hanlon P, McEwen J, Carey L, *et al.* Health checks and coronary risk: further evidence from a randomised controlled trial. Bmj. 1995;**311**:1609–13. doi:10.1136/bmj.311.7020.1609

19 Paterson JM, Llewellyn-Thomas HA, Naylor CD. Using disease risk estimates to guide risk factor interventions: Field test of a patient workbook for self-assessing coronary risk. *Heal Expect* 2002;**5**:3–15. doi:10.1046/j.1369-6513.2002.00148.x

20 Sheridan SL, Shadle J, Simpson RJ, *et al.* The impact of a decision aid about heart disease prevention on patients’ discussions with their doctor and their plans for prevention: a pilot randomized trial. *BMC Health Serv Res* 2006;**6**:121. doi:10.1186/1472-6963-6-121

21 Weymiller AJ, Montori VM, Jones LA, *et al.* Helping patients with type 2 diabetes mellitus make treatment decisions: statin choice randomized trial. *Arch Intern Med* 2007;**167**:1076–82. doi:10.1001/archinte.167.10.1076

22 Edelman D, Oddone EZ, Liebowitz RS, *et al.* A Multidimensional Integrative Medicine Intervention to Improve Cardiovascular Risk. 2006;**27705**:728–34. doi:10.1111/j.1525-1497.2006.0495.x

23 Krones T, Keller H, Sadowski E, *et al.* Absolute Cardiovascular Disease Risk and Shared Decision Making in Primary Care : A Randomized Controlled Trial. *Ann Fam Med* 2008;**6**:218–27. doi:10.1370/afm.854.INTRODUCTION

24 Lalonde L, O’Connor a, Drake E, *et al.* Development and preliminary testing of a patient decision aid to assist pharmaceutical care in the prevention of cardiovascular disease. *Pharmacotherapy* 2004;**24**:909–22.

25 Lovibond SH, Birrell PC, Langeluddecke P. Changing coronary heart disease risk-factor status: The effects of three behavioral programs. *J Behav Med* 1986;**9**:415–37. doi:10.1007/BF00845131

26 Muir J, Mant D, Jones L, *et al.* Effectiveness of health checks conducted by nurses in primary care: results of the OXCHECK study after one year. Imperial Cancer Research Fund OXCHECK Study Group. *BMJ* 1994;**308**:308–12.http://www.bmj.com/cgi/doi/10.1136/bmj.308.6924.308

27 Imperial Cancer Research Fund OSG. Effectiveness of health checks conducted by nurses in primary care: final results of the OXCHECK study. *Bmj* 1995;**130**:1099–104. doi:10.1136/bmj.308.6924.308

28 Turnbull DA, Beilby JJ, Ziaian T, *et al.* Disease Management for Hypertension. *Dis Manag Heal Outcomes* 2006;**14**:27–35. doi:10.2165/00115677-200614010-00004

29 Williams GC, McGregor H, Sharp D, *et al.* A self-determination multiple risk intervention trial to improve smokers’ health. *J Gen Intern Med* 2006;**21**:1288–94. doi:10.1111/j.1525-1497.2006.00621.x

30 Family Heart Study Group. Randomised controlled trial evaluating cardiovascular screening and intervention in general practice: principal results of British family heart study. Family Heart Study Group. *BMJ* 1994;**308**:313–20. doi:10.1136/bmj.308.6924.313

31 Emmett CL, Montgomery A a, Peters TJ, *et al.* Three-year follow-up of a factorial randomised controlled trial of two decision aids for newly diagnosed hypertensive patients. *Br J Gen Pract* 2005;**55**:551–3./pmc/articles/PMC1472777/?report=abstract

32 Montgomery AA, Fahey T, Peters TJ. A factorial randomised controlled trial of decision analysis and an information video plus leaflet for newly diagnosed hypertensive patients. *Br J Gen Pract* 2003;**53**:446–53.http://www.ncbi.nlm.nih.gov/pubmed/12939889

33 Wister A, Loewen N, Kennedy-Symonds H, *et al.* One-year follow-up of a therapeutic lifestyle intervention targeting cardiovascular disease risk. *CMAJ* 2007;**177**:859–65. doi:10.1503/cmaj.061059

34 Asimakopoulou KG, Fox C, Spimpolo J, *et al.* The impact of different time frames of risk communication on Type 2 diabetes patients’ understanding and memory for risk of coronary heart disease and stroke. *Diabet Med* 2008;**25**:811–7. doi:10.1111/j.1464-5491.2008.02473.x

35 Charlson ME, Peterson JC, Boutin-Foster C, *et al.* Changing health behaviors to improve health outcomes after angioplasty: a randomized trial of net present value versus future value risk communication. *Health Educ Res* 2008;**23**:826–39. doi:10.1093/her/cym068

36 Straus SE. Individualizing Treatment Decisions. *Eval Health Prof* 2002;**25**:210–24. doi:10.1177/016327870202500206

37 Meland E, Laerum E, Ulvik RJ. Effectiveness of two preventive interventions for coronary heart disease in primary care. *Scand J Prim Health Care* 1997;**15**:57–64.http://www.ncbi.nlm.nih.gov/pubmed/9101627

38 Ketola E, Mäkelä M, Klockars M. Individualised multifactorial lifestyle intervention trial for high-risk cardiovascular patients in primary care. *Br J Gen Pract* 2001;**51**:291–4.

39 MRFIT. Multiple risk factor intervention trial. Risk factor changes and mortality results. Multiple Risk Factor Intervention Trial Research Group. *JAMA* 1982;**248**:1465–77.http://www.ncbi.nlm.nih.gov/pubmed/7050440

40 Kornitzer M, Rose G. WHO European collaborative trial of multifactorial prevention of coronary heart disease. *Prev Med (Baltim)* 1985;**14**:272–8. doi:10.1016/0091-7435(85)90056-8

41 Hussein HM, Harris-Lane P, Abdelmoula MM, *et al.* Accuracy of self-perception of cardiovascular risk in the community. *J Vasc Interv Neurol* 2008;**1**:106–12.http://www.pubmedcentral.nih.gov/articlerender.fcgi?artid=3317327&tool=pmcentrez&rendertype=abstract

42 Persell SD, Lloyd-Jones DM, Friesema EM, *et al.* Electronic health record-based patient identification and individualized mailed outreach for primary cardiovascular disease prevention: a cluster randomized trial. *J Gen Intern Med* 2013;**28**:554–60. doi:10.1007/s11606-012-2268-1

43 Price HC, Griffin SJ, Holman RR. Impact of personalized cardiovascular disease risk estimates on physical activity-a randomized controlled trial. *Diabet Med* 2011;**28**:363–72. doi:10.1111/j.1464-5491.2010.03212.x

44 Qureshi N, Armstrong S, Dhiman P, *et al.* Effect of adding systematic family history enquiry to cardiovascular disease risk assessment in primary care: a matched-pair, cluster randomized trial. *Ann Intern Med* 2012;**156**:253–62. doi:10.7326/0003-4819-156-4-201202210-00002

45 Bucher HC, Rickenbach M, Young J, *et al.* Randomized trial of a computerized coronary heart disease risk assessment tool in HIV-infected patients receiving combination antiretroviral therapy. *Antivir Ther* 2010;**15**:31–40. doi:10.3851/IMP1475

46 Benner JS, Cherry SB, Erhardt L, *et al.* Rationale, design, and methods for the risk evaluation and communication health outcomes and utilization trial (REACH OUT). *Contemp Clin Trials* 2007;**28**:662–73. doi:10.1016/j.cct.2007.03.004

47 Bertoni AG, Bonds DE, Chen H, *et al.* Impact of a multifaceted intervention on cholesterol management in primary care practices: guideline adherence for heart health randomized trial. *Arch Intern Med* 2009;**169**:678–86. doi:10.1001/archinternmed.2009.44

48 Cobos A, Vilaseca J, Asenjo C, *et al.* Cost Effectiveness of a Clinical Decision Support System Based on the Recommendations of the European Society of Cardiology and Other Societies for the Management of Hypercholesterolemia. *Dis Manag Heal Outcomes* 2005;**13**:421–32. doi:10.2165/00115677-200513060-00007

49 Denig P, Schuling J, Haaijer-Ruskamp F, *et al.* Effects of a patient oriented decision aid for prioritising treatment goals in diabetes: pragmatic randomised controlled trial. *BMJ* 2014;**349**:g5651. doi:10.1016/j.apergo.2011.09.004

50 Eaton CB, Parker DR, Borkan J, *et al.* Translating cholesterol guidelines into primary care practice: a multimodal cluster randomized trial. *Ann Fam Med* 2011;**9**:528–37. doi:10.1370/afm.1297

51 Engberg M, Christensen B, Karlsmose B, *et al.* General health screenings to improve cardiovascular risk profiles: a randomized controlled trial in general practice with 5-year follow-up. *J Fam Pract* 2002;**51**:546–52.http://www.ncbi.nlm.nih.gov/pubmed/12100779

52 Holt TA, Thorogood M, Griffiths F, *et al.* Automated electronic reminders to facilitate primary cardiovascular disease prevention: randomised controlled trial. *Br J Gen Pract* 2010;**60**:e137-43. doi:10.3399/bjgp10X483904

53 Jørgensen T, Jacobsen RK, Toft U, *et al.* Effect of screening and lifestyle counselling on incidence of ischaemic heart disease in general population: Inter99 randomised trial. *BMJ* 2014;**348**:g3617. doi:10.1016/j.ypmed.2016.08.016

54 Koelewijn-van Loon MS, van der Weijden T, Ronda G, *et al.* Improving lifestyle and risk perception through patient involvement in nurse-led cardiovascular risk management: a cluster-randomized controlled trial in primary care. *Prev Med (Baltim)* 2010;**50**:35–44. doi:10.1016/j.ypmed.2009.11.007

55 Lopez-Gonzalez AA, Aguilo A, Frontera M, *et al.* Effectiveness of the Heart Age tool for improving modifiable cardiovascular risk factors in a Southern European population: a randomized trial. *Eur J Prev Cardiol* 2015;**22**:389–96. doi:10.1177/2047487313518479

56 Mann DM, Ponieman D, Montori VM, *et al.* The Statin Choice decision aid in primary care: a randomized trial. *Patient Educ Couns* 2010;**80**:138–40. doi:10.1016/j.pec.2009.10.008

57 Peiris D, Usherwood T, Panaretto K, *et al.* Effect of a computer-guided, quality improvement program for cardiovascular disease risk management in primary health care: the treatment of cardiovascular risk using electronic decision support cluster-randomized trial. *Circ Cardiovasc Qual Outcomes* 2015;**8**:87–95. doi:10.1161/CIRCOUTCOMES.114.001235

58 Perestelo-Pérez L, Rivero-Santana A, Boronat M, *et al.* Effect of the statin choice encounter decision aid in Spanish patients with type 2 diabetes: A randomized trial. *Patient Educ Couns* 2016;**99**:295–9. doi:10.1016/j.pec.2015.08.032

59 Persell SD, Brown T, Lee JY, *et al.* Individualized Risk Communication and Outreach for Primary Cardiovascular Disease Prevention in Community Health Centers: Randomized Trial. *Circ Cardiovasc Qual Outcomes* 2015;**8**:560–6. doi:10.1161/CIRCOUTCOMES.115.001723

60 Sheridan SL, Draeger LB, Pignone MP, *et al.* A randomized trial of an intervention to improve use and adherence to effective coronary heart disease prevention strategies. *BMC Health Serv Res* 2011;**11**:331. doi:10.1186/1472-6963-11-331

61 Soureti A, Murray P, Cobain M, *et al.* Web-based risk communication and planning in an obese population: exploratory study. *J Med Internet Res* 2011;**13**:e100. doi:10.2196/jmir.1579

62 Turner BJ, Hollenbeak CS, Liang Y, *et al.* A randomized trial of peer coach and office staff support to reduce coronary heart disease risk in African-Americans with uncontrolled hypertension. *J Gen Intern Med* 2012;**27**:1258–64. doi:10.1007/s11606-012-2095-4

63 Vagholkar S, Zwar N, Jayasinghe UW, *et al.* Influence of cardiovascular absolute risk assessment on prescribing of antihypertensive and lipid-lowering medications: a cluster randomized controlled trial. *Am Heart J* 2014;**167**:28–35. doi:10.1016/j.ahj.2013.10.002

64 Webster R, Li SC, Sullivan DR, *et al.* Effects of internet-based tailored advice on the use of cholesterol-lowering interventions: a randomized controlled trial. *J Med Internet Res* 2010;**12**:e42. doi:10.2196/jmir.1364

65 Welschen LMC, Bot SDM, Kostense PJ, *et al.* Effects of cardiovascular disease risk communication for patients with type 2 diabetes on risk perception in a randomized controlled trial: the @RISK study. *Diabetes Care* 2012;**35**:2485–92. doi:10.2337/dc11-2130

66 Zullig LL, Sanders LL, Shaw RJ, *et al.* A randomised controlled trial of providing personalised cardiovascular risk information to modify health behaviour. *J Telemed Telecare* 2014;**20**:147–52. doi:10.1177/1357633X14528446

67 Pieper D, Antoine S-L, Mathes T, *et al.* Systematic review finds overlapping reviews were not mentioned in every other overview. *J Clin Epidemiol* 2014;**67**:368–75.http://www.embase.com/search/results?subaction=viewrecord&from=export&id=L372497994
